# Impact of free *N*^ε^-carboxymethyllysine, its precursor glyoxal and AGE-modified BSA on serotonin release from human parietal cells in culture

**DOI:** 10.1039/c8fo01045e

**Published:** 2018-07-04

**Authors:** Ann-Katrin Holik, Verena Stöger, Kathrin Hölz, Mark M. Somoza, Veronika Somoza

**Affiliations:** a Department of Physiological Chemistry , Faculty of Chemistry , University of Vienna , Althanstraße 14 , 1090 Vienna , Austria . Email: Veronika.Somoza@univie.ac.at ; Fax: +43 1 4277 9706 ; Tel: +43 1 4227 70601; b Christian Doppler Laboratory for Bioactive Aroma Compounds , Faculty of Chemistry , University of Vienna , Althanstraße 14 , 1090 Vienna , Austria; c Department of Inorganic Chemistry , Faculty of Chemistry , University of Vienna , Althanstraße 14 , 1090 Vienna , Austria

## Abstract

Advanced glycation end products (AGEs) are frequently encountered in a western diet, in addition to their formation *in vivo*. *N*-Epsilon-carboxymethyllysine (CML), one of the chemically diverse compounds formed in the reaction between reducing carbohydrates and amines, is often used as a marker of advanced glycation, and has been shown to stimulate serotonin release from cells representing the central (SH-SY5Y cells) and the peripheral (Caco-2 cells) serotonin system *in vitro*. Here, we investigated the effect of glyoxal, free CML, and protein-linked AGE-BSA on serotonin release from human gastric tumour cells, which originate from an adenocarcinoma of the stomach and have recently been shown to be capable of serotonin synthesis and release. Microarray experiments showed both CML and glyoxal to alter genes associated with serotonin receptors. Furthermore, treatment with glyoxal resulted in a small change in RAGE expression while CML did not alter its expression. On a functional level, treatment with 500 μM CML increased extracellular serotonin content by 341 ± 241%, while treatment with 1 mg mL^–1^ AGE-BSA led to a reduction by 49 ± 11% compared to non-treated cells. The CML-induced serotonin release was reduced by the HTR3 antagonist granisetron. Incubation with the RAGE antagonist FPS-ZM1 abolished the effect of AGE-BSA on serotonin release, while no impact on CML-induced serotonin release was observed. Furthermore, treatment with 5 mM CML stimulated proton secretion as a functional outcome measure, assessed using a pH sensitive dye. Taken together, these results indicate a likely HTR3-mediated, RAGE-independent effect of free CML on serotonin release and a RAGE-dependent mechanism for the protein linked AGE-BSA.

## Abstract 2

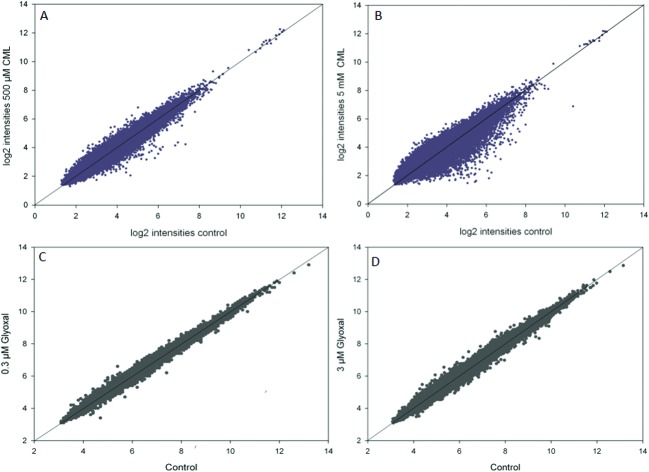
The advanced glycation endproduct CML, often encountered in a Western diet, increases serotonin release from cultured parietal cells, while a protein-linked AGE showed the opposite effect.

## Introduction

Advanced glycation end products (AGEs) are formed by thermal processing of foods at high temperatures. In addition to the formation of AGEs, the high thermal heat treatment encountered in many typical western diets, may alter other food properties such as energy density. A study by Birlouez-Aragon *et al.*,^1^ testing a standard diet high in AGEs and a steamed diet low in AGEs, reported the standard diet to have a higher energy density compared to the steamed diet. In addition, consumption of the standard diet high in AGEs resulted in a higher total energy intake compared to the low AGE meals.^1^ By regular ingestion as part of a typical western diet, AGEs partially enter systemic circulation.^1^–^4^ *N*^ε^-Carboxymethyllysine (CML) is a stable and well-characterized AGE which is often used as a marker for AGEs in foods^5^–^7^ and has been detected in human plasma, centring at a concentration around 3 μM.^8^ In addition to CML, one of its precursors, glyoxal, has been identified in human plasma at concentrations around 0.3 μM.^9^ Both forms, protein-bound and free CML, have been investigated in several studies, the latter to lesser extent. A study on HEK-293 cells indicated protein-linked CML and free CML to induce p38 MAPK activation. This study used HEK-293 cells only expressing the extracellular domain of RAGE, and thus lacking the ability to intracellularly transduce RAGE-mediated signals. Zill *et al.*^10^ suggested a RAGE-mediated pathway for both protein-bound and free CML. A RAGE-depended pathway has also been described by Schmid *et al.*^11^ in experiments on the expression of heat shock proteins in which Caco-2 cells were incubated with casein-linked or free CML. Furthermore, free CML is known to stimulate serotonin release from cells representing central and peripheral serotonin.^12^,^13^ Serotonin is an important neurotransmitter in the regulation of feeding and satiety.^14^ The activation of several serotonin receptors has been described to increase or decrease food intake, for some of which alterations in gene expression have been shown after CML treatment.^13^ Several amino acids also influence satiety; a study on adult male Sprague-Dawley rats on an amino acid supplemented diet indicated CML's precursor amino acid, lysine, increases satiety.^15^

While the effects of AGEs have been studied in several cell lines and their accumulation in diverse tissues *in vivo* have been assessed, less information is available on the potential effects on the stomach. In a study by Morioka *et al.*,^16^ positive Glycer-AGE immunostaining was observed in the stomachs of healthy rats. Argirova and colleagues^17^ assessed the effects of water-soluble Maillard reaction products (MRPs), derived from different model systems, on the mechanical activity of the gastric smooth muscle from rats. Several of the compounds applied in this work led to contractions, among which the reaction products formed between glucose and arginine produced the strongest effect. Furthermore, the authors reported the precursor carbohydrates and amino acids not to have an impact on the mechanical gastric smooth muscle activity. AGEs have also been reported to interfere with the expression of gastric smooth muscle contractile markers involving RAGE and NF-κB in streptozotocin-induced diabetic rats.^18^ In a study by Wang *et al.*^19^ gastric muscle tissue from patients undergoing gastric surgery was collected and the expression of contractile proteins correlated with the CML levels of individuals in a diabetic and a control group. Decreased expression of contractile proteins and phosphorylation levels of proteins involved in the contractile regulation were found in the diabetic group. In addition, this group showed increased CML concentrations in the gastric muscle layer. Fujiwara *et al.*^20^ have shown dietary uptake of compounds possessing a catechol group to enhance the formation of CML. In their experiments, CML was shown to accumulate at the surface area of the gastric epithelial cells in the stomach of streptozotocin-induced diabetic mice which received 500 mg kg^–1^ day^–1^ epicatechin.

The human gastric tumour cell line (HGT-1), derived from a poorly differentiated adenocarcinoma of the stomach, was introduced by Laboisse and co-workers in 1982.^21^ This cell line has since been shown to possess the transporters required in gastric acid release^22^ and consequently been used in the determination of a test compound's effect on proton secretion using the fluorescent properties of a pH sensitive dye.^23^,^24^ As little information is available on the effects of dietary derived AGEs on the stomach and HGT-1 cells have been shown to release serotonin upon stimulation^25^ (unpublished data), we used this cell line in assessing the effects of free CML, its precursor glyoxal and protein-linked AGE-BSA on serotonin release. Further, we investigated a potential HTR3 link as serotonin has been suggested to alter gastric acid release *via* HTR3 receptors.^26^

## Materials and methods

### Materials

Synthetic CML, free from proteins and hydrochloride, was obtained from Iris Biotech (Markredwitz, Germany). AGE-BSA and FPS-ZM1 were ordered from Merck Millipore. Glyoxal and all other reagents required were ordered from Sigma-Aldrich (Austria).

### Cell culture

HGT-1 cells were received from C. Laboisse (Laboratory of Pathological Anatomy, France) and cultured in Dulbecco's modified Eagle medium (DMEM). The medium was supplemented with 10% fetal bovine serum, 4 mM l-glutamine and 1% penicillin/streptomycin. Cells were maintained at 37 °C in a humidified incubator at 5% CO_2_ and sub-cultured at 90% confluence.

### Viability assay

Negative effects of any of the treatments on metabolic activity was excluded by MTT-assay (3-(4,5-dimethylthiazolyl-2)-2,5-diphenyltetrazolium bromide). Briefly, HGT-1 cells were seeded in 96-well plates at a density of 100 000 cells per well and left to settle overnight. Next, the medium was aspirated and test compounds added dissolved in either Krebs-Ringer HEPES buffer or serum-free medium. After incubation for 20 to 90 min, the compounds were removed and MTT working solution (diluted in serum-free medium to a final concentration of 1 mg mL^–1^) added. After formation of purple formazan crystals (approx. 15 min), the MTT-working solution was replaced by DMSO and the absorbance recorded at 570 nm.

### Gene expression

#### DNA microarrays

DNA microarrays were prepared using a maskless synthesis approach as described previously.^27^ Two microarrays were synthesized at once as described by Sack *et al.*^28^ HGT-1 cells were seeded in 6-well plates prior to incubation with either CML (500 μM, 5 mM) or glyoxal (0.3 μM, 3 μM) for 90 min in serum-free medium.^12^ RNA was isolated using the RNeasy Mini Kit (Qiagen). To verify no DNA co-purification occurred and to check the integrity of the isolated RNA, agarose gel electrophoresis was carried out in addition to determining *A*_260/230_ and *A*_260/280_ ratios photometrically. Using the SuperScript III Kit (Invitrogen) and random nonamer primers carrying a Cy3-tag (Tebu Bio), 10 μg RNA were reverse transcribed^29^ and cleaned up using the QIAquick PCR purification kit (Qiagen). The hybridization solution consisted of the generated Cy3-tagged cDNA, synthetic quality controls (25mer, 2× 60mer), BSA, herring sperm DNA and MES buffer. After allowing the DNA to hybridize for 20 h at 42 °C under constant rotation in the dark, the microarrays were washed. Afterwards, the microarrays were scanned using an Axon GenePix 4400A device (Molecular Devices). The scanned images were analyzed using NimbleScan 2.1 (NimbleGen) and normalized using robust multichip analysis. After extracting normalized intensities, fold-changes of CML/glyoxal-treatment to the untreated control were calculated and genes reaching either 1.2 up- or down-regulation input into the DAVID pathway analysis software (; http://david.abcc.ncifcrf.gov), which was used for identifying potential candidates^30^ for further analysis.

### qPCR

HGT-1 cells were seeded into 24-well plates at a density of 1.5 × 10^5^ cells per well 24 h prior incubation with CML (500 μM) or glyoxal (3 μM) for 15–90 min. RNA was isolated using the PeqGold Total RNA kit (Peqlab), a spin-column based kit which employs DNA-removing columns in the first isolation step. RNA isolation was followed by reverse transcription using the high capacity cDNA kit (Life Technology). SYBR green master mix (Life Technology) was used in the qPCR reaction on a StepOnePlus device (Applied biosystems). The sequences of all primers used are shown in Table 1.^13^,^31^ Data analysis was carried out using LinReg v2013.0 32,33 and normalized to the expression of TBP (TATA-box binding protein) and PPIA (peptidylprolyl isomerase A).

**Table 1:** Sequences of the primers used in qPCR

Target	Forward primer	Reverse primer	Product length (bp)
TBP	CCCGAAACGCCGAATATAATCC	GACTGTTCTTCACTCTTGGCTC	130
PPIA	CCACCAGATCATTCCTTCTGTAGC	CTGCAATCCAGCTAGGCATGG	144
RAGE	ACTACCGAGTCCGTGTCTACC	GGAACACCAGCCGTGAGTT	79

### Serotonin release

The serotonin concentration after stimulation with CML (5–5000 μM), glyoxal (0.03–30 μM), lysine (5–5000 μM), BSA (0.5–1 mg mL^–1^) or AGE-BSA (0.5–1 mg mL^–1^) was determined by ELISA (serotonin high sensitive, DLD Diagnostika) as described previously^34^ with the following modification: the incubation time was extended from 5 min to 20 min.

### CML concentration

The CML concentration of AGE-BSA and BSA was determined after hydrolysis as described by Teerlink *et al.*^8^ Briefly, the protein (0.2–4 mg) was dissolved in water and sodium borohydride (100 mM in 200 mM sodium borate buffer pH 9.2) added. The mixture was incubated in a Pyrex glass reaction tube with a Teflon-lined screw cap for 2 h at room temperature before TFA (200 g L^–1^) was added and the protein pelleted by centrifugation at 2000*g* for 10 min. The supernatant was removed prior to addition of another aliquot of TFA (100 g L^–1^) and a further centrifugation step. After the removal of all TFA, HCl (6 M) and internal standard (^13^C-CML, kindly provided by Dr John Baynes) were added. After heating the mixture to 110 °C for 20 h, the acid was evaporated and the solid material dissolved in LC-MS/MS starting eluent. The starting conditions were 90% acetonitrile and 10% of a 6 mM ammonium formate buffer (pH 2.2). Gradient component A was acetonitrile supplemented with 6 mM ammonium formate and gradient component B was aqueous 6 mM ammonium formate buffer (pH 2.2). The following gradient was set: 0–2 min 90% B, 3–5 min 87% B, 9–15 min 30% B, 20–30 min 90% B. The flow rate was set to 0.4 mL min^–1^. The analysis was carried out on a Shimadzu 8040 LC-M/MS using a Luna 3 μm HILIC 200 A column (100 × 3 mm, Phenomenex) equipped with a HILIC 4 × 2.00 mm SecurityGuard pre-column. A total of 10 μL sample and standards was injected and a valve used for only allowing effluent between 9 and 15 min of the run time to enter the MS. The MS interface settings were as follows: nebulizing gas flow 3 L min^–1^, DL temperature 170 °C, heat block temperature 350 °C, drying gas flow 13 L min^–1^. Lysine, CML and ^13^C-CML were detected in positive MRM mode. Table 2 shows the corresponding settings. The transition from 205 *m*/*z* to 83 *m*/*z* was used in the quantification of CML. The calibration range was 100–10 000 ng mL^–1^ for CML and 250–10 000 ng mL^–1^ for lysine.

**Table 2:** MRM settings used for the quantification of CML in AGE-BSA and BSA

Analyte	Precursor (*m*/*z*)	Product (*m*/*z*)	Dwell time (ms)	Q1 pre bias (V)	CE	Q3 pre bias (V)
CML	205	84	400	–12	–19	–13
CML	205	130	200	–16	–14	–20
Lys	147	84	100	–12	–10	–13
^13^C-CML	207	130	100	–16	–14	–20

### Proton secretion

HGT-1 cells were seeded in a 96-well plate (FluoroNunc™ F96 MicroWell™, VWR) at a density of 100 000 cells per well. After allowing the cells to settle for 24 h, the cells were washed with Krebs-HEPES buffer (10 mM HEPES, 11.7 mM d-glucose, 4.7 mM KCl, 130 mM NaCl, 1.3 mM CaCl_2_, 1.2 mM MgSO_4_, and 1.2 mM KH_2_PO_4_, adjusted to pH 7.4 with 5 M KOH). Then, the cells were stained with the pH-sensitive fluorescent dye 1,5-carboxy-seminaphtorhodafluor acetoxymethylester (3 μM, SNARF-1-AM, Life Technologies) for 30 min before washing twice and adding test compound (100 μL, 500 μM CML, 5 mM CML) or standard (pH calibration pH 7.2–8.2, potassium, buffer calibration solution: 20 mM HEPES, 18 mM d-glucose, 110 mM KCl, 20 mM NaCl, 1 mM CaCl_2_, 1 mM MgSO_4_). The fluorescence was measured using an Infinite 200 Pro Plate reader (Tecan) with the excitation wavelength set to 488 nm and the emission recorded at 580 nm and 640 nm for 30 min. The intracellular proton index (IPX) was calculated using the pH calibration generated and the emission ratio and performing a log_2_ transformation.

### Statistical analysis

All data are displayed as average with standard deviation as stated in the corresponding table or figure legend. Data shown as fold-change (denominated T/C in the figures) were calculated in relation to the control which was set to 1 or 100%. For statistical analysis SigmaPlot 11 was used.

## Results

### Cell viability

None of the treatments applied had any negative effects on cellular viability as determined by MTT-assay (data not shown).

### Gene expression

**Fig. 1:**
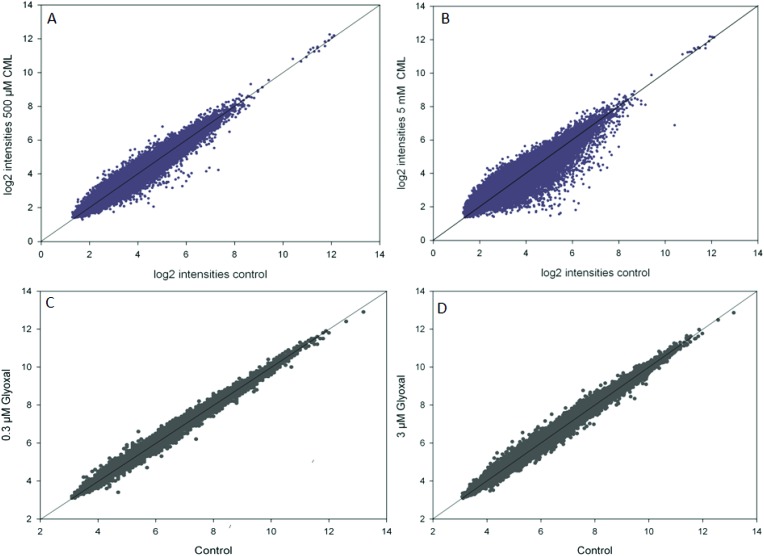
Scatterplots of log_2_ transformed intensities of microarray data for control and treatment (A, B: CML; C, D: glyoxal), data are shown as average of at least three independent biological replicates.

In the microarray experiments, HGT-1 cells were treated with CML (500 μM, 5 mM) or glyoxal (0.3 μM, 3 μM) for 90 min. The scatterplots of log_2_ intensities of treatment *vs.* control (Fig. 1) show a stronger impact on gene regulation by treatment with CML (1A, B) compared to glyoxal treatment (1C, D) as illustrated by a wider distribution of probes. A solid diagonal line represents a fold-change of 1, *i.e.* no difference between control and treatment. The scatterplots suggest treatment with 5 mM CML to have the biggest impact of the conditions tested. In the next step, probes reaching a fold-change of 1.2 up- or down-regulation were further assessed using the free online tool DAVID. The first cluster obtained after DAVID analysis is shown in Table 3 for CML (A: 500 μM, B: 5 mM) and in Table 4 for glyoxal (A: 0.3 μM, B: 3 μM). All four clusters were found to contain serotonin receptors, reaching similar enrichment scores each.

**Table 3:** Microarray data after treatment with 500 μM CML (A) or 5 mM CML (B), showing the cluster with the highest enrichment score after DAVID pathway analysis. Probes reaching a fold-change below 0.8 or above 1.2 in all three biological replicates were used in the analysis

	*p*-Value	Benjamini
A: 500 μM CML		
Serotonin receptor signalling pathway	1.7 × 10^–8^	8.0 × 10^–5^
G-protein coupled serotonin receptor activity	4.4 × 10^–6^	6.5 × 10^–3^
Serotonin binding	7.1 × 10^–6^	5.3 × 10^–3^
5-Hydroxytryptamine receptor family	4.6 × 10^–5^	1.1 × 10^–1^
Neurotransmitter receptor activity	8.5 × 10^–5^	2.5 × 10^–2^
Behaviour	6.1 × 10^–4^	3.6 × 10^–2^
Regulation of behaviour	8.0 × 10^–4^	5.3 × 10^–1^
Release of sequestered calcium ion into cytosol	1.5 × 10^–3^	5.1 × 10^–1^
Serotonergic synapse	1.1 × 10^–2^	2.1 × 10^–1^
Vasoconstriction	2.0 × 10^–2^	8.6 × 10^–1^
Adenylate cyclase-inhibiting G-protein coupled receptor signalling pathway	3.6 × 10^–2^	9.2 × 10^–1^
Positive regulation of phosphatidylinositol biosynthetic process	5.0 × 10^–2^	9.5 × 10^–1^

B: 5 mM CML		
SM01381	1.1 × 10^–10^	4.3 × 10^–8^
Neuroactive ligand–receptor interaction	1.4 × 10^–9^	3.8 × 10^–7^
Receptor	5.2 × 10^–8^	1.3 × 10^–5^
Lipid moiety-binding region: S-palmitoyl cysteine	1.3 × 10^–4^	1.1 × 10^–1^
G-protein coupled receptor	4.3 × 10^–4^	1.9 × 10^–2^
Transducer	2.0 × 10^–3^	6.3 × 10^–2^
G-protein coupled receptor, rhodopsin-like	4.9 × 10^–3^	9.6 × 10^–1^
GPCR, rhodopsin-like, 7TM	8.3 × 10^–3^	9.7 × 10^–1^
G-protein coupled receptor signaling pathway	2.9 × 10^–1^	1.0
G-protein coupled receptor activity	7.4 × 10^–1^	1.0

**Table 4:** Microarray data after treatment with 0.3 μM glyoxal (A) or 3.0 μM glyoxal (B), showing the cluster with the highest enrichment score after DAVID pathway analysis. Probes reaching a fold-change below 0.8 or above 1.2 in all three biological replicates were used in the analysis

	*p*-Value	Benjamini
A: 0.3 μM glyoxal		
Neuroactive ligand–receptor interaction	1.4 × 10^–11^	1.3 × 10^–9^
SM01381	1.7 × 10^–8^	1.1 × 10^–6^
Receptor	8.2 × 10^–8^	1.5 × 10^–5^
G-protein coupled receptor	7.9 × 10^–7^	7.3 × 10^–5^
Transducer	1.9 × 10^–6^	1.2 × 10^–4^
Topological domain: extracellular	3.6 × 10^–6^	1.2 × 10^–3^
G protein-coupled receptor, rhodopson-like	8.3 × 10^–5^	2.2 × 10^–2^
GPCR, rhodopsin-like, 7TM	1.0 × 10^–4^	1.4 × 10^–2^
Transmembrane helix	2.0 × 10^–4^	9.4 × 10^–3^
Transmembrane	2.2 × 10^–4^	8.1 × 10^–3^
Topological domain: cytoplasmic	2.7 × 10^–4^	4.5 × 10^–2^
Integral component of plasma membrane	3.8 × 10^–4^	5.8 × 10^–2^
Glycosylation site: N-linked	1.1 × 10^–3^	9.0 × 10^–2^
Cell membrane	1.3 × 10^–3^	3.0 × 10^–2^
Transmembrane region	2.0 × 10^–3^	1.3 × 10^–1^
Plasma membrane	5.6 × 10^–3^	3.6 × 10^–1^
Glycoprotein	5.9 × 10^–3^	9.6 × 10^–2^
Membrane	6.0 × 10^–3^	8.9 × 10^–2^
Disulfide bond	8.2 × 10^–3^	1.1 × 10^–1^
Disulfide bond	9.1 × 10^–3^	4.0 × 10^–1^
Integral component of membrane	7.8 × 10^–2^	8.4 × 10^–1^
G-protein coupled receptor activity	1.8 × 10^–1^	9.7 × 10^–1^

B: 3.0 μM glyoxal		
G-protein coupled serotonin receptor activity	4.6 × 10^–7^	1.6 × 10^–4^
Serotonin receptor signaling pathway	9.6 × 10^–6^	5.9 × 10^–3^
Serotonergic synapse	1.0 × 10^–4^	8.2 × 10^–3^

The mRNA expression of RAGE was analysed by qPCR after incubation with 500 μM CML or 3 μM glyoxal for 15, 30, 60 or 90 min (Table 5). Treatment with glyoxal resulted in alterations in RAGE expression at 30 min (0.73 ± 0.15, *p* < 0.05). The other incubations did not yield any changes in RAGE expression compared to the control.

**Table 5:** RAGE mRNA expression assessed by qPCR after treatment with glyoxal (3 μM) or CML (500 μM) for 15–90 min, data are shown as average and standard deviation of at least three independent biological replicates, statistics: two-way ANOVA *vs.* control, * = *p* < 0.05

	90 min	60 min	30 min	15 min
Control	1.00 ± 0.06	1.00 ± 0.04	1.00 ± 0.08	1.00 ± 0.07
CML	1.01 ± 0.09	0.97 ± 0.02	0.93 ± 0.14	0.79 ± 0.08
Glyoxal	1.01 ± 0.05	0.82 ± 0.13	0.73 ± 0.15*	1.02 ± 0.24

### Serotonin release

**Fig. 2:**
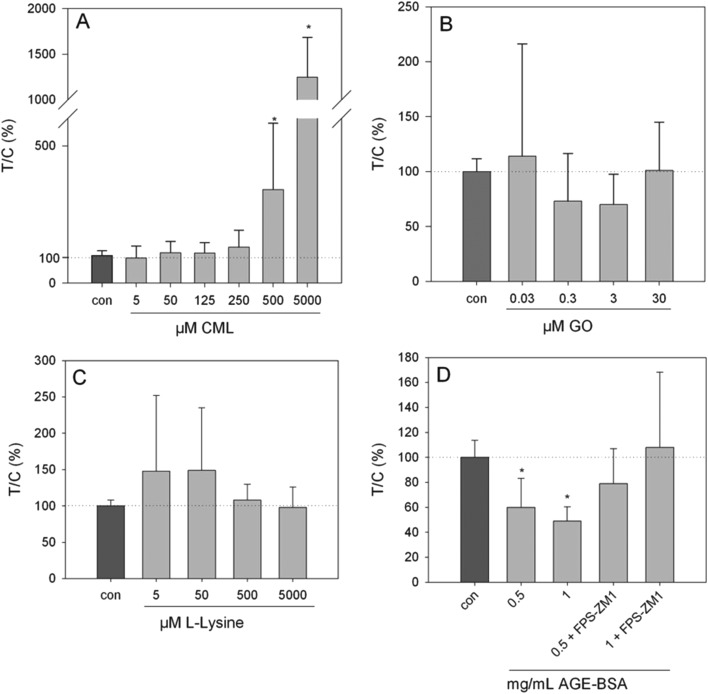
Serotonin release from HGT-1 cells after 20 min treatment with CML (A, 5 μM–5 mM), glyoxal (B, 0.03–30 μM), lysine (C, 5 μM–5 mM) or AGE-BSA (D, 0.5–1 mg mL^–1^) determined by ELISA, data are shown as average with corresponding standard deviation from at least three independent biological replicates, statistics: Kruskal–Wallis one-way analysis of variance on ranks, multiple comparisons *vs.* control (Dunn's method).

**Fig. 3:**
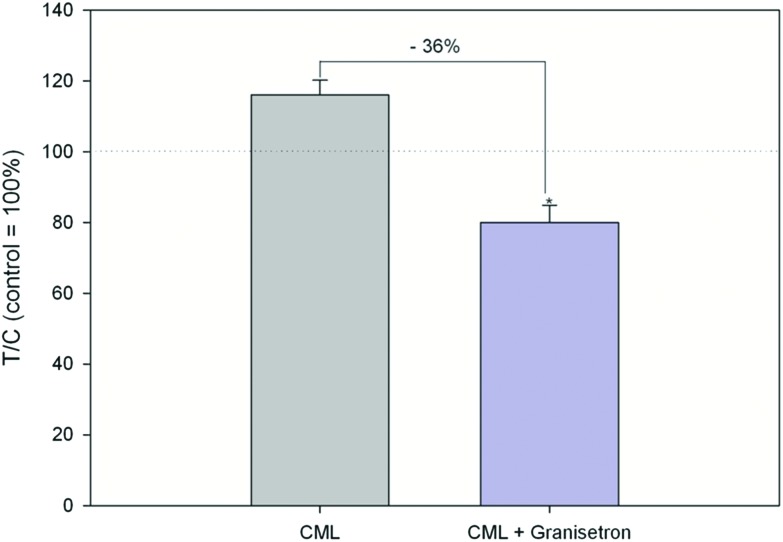
Serotonin release after treatment with 500 μM CML or co-incubation of HTR3 antagonist granisetron (10 μM) and 500 μM CML, data are shown as average with corresponding standard deviation and calculated in relation to CML treatment (set to 100%), Statistics: Mann–Whitney rank sum test CML *vs.* control (*p* = 0.002; not depicted in graph), *t*-test CML *vs.* granisetron (* = *p* < 0.001).

Exposure of HGT-1 cells to 500 μM or 5 mM CML increased the extracellular serotonin concentration to 341 ± 241% (*p* < 0.05, Fig. 3A) and 1247 ± 438% (*p* < 0.05, Fig. 2A), respectively, while glyoxal did not induce changes in the serotonin levels at any of the concentrations tested (Fig. 2B). The effect of 500 μM CML was not changed by co-incubation with the RAGE antagonist FPS-ZM1 (100.5 ± 39%, CML set to 100%, *n* = 7). Co-incubation with the HTR3 inhibitor granisetron decreased the CML-induced serotonin release by 36% (Fig. 3). Incubation with AGE-BSA at concentrations of 0.5 or 1 mg mL^–1^ decreased the serotonin concentration in the extracellular supernatant to 60 ± 23% (*p* < 0.05, Fig. 2D) and 49 ± 11 (*p* < 0.05, Fig. 2D), respectively. These effects were abolished in a co-incubation experiment with FPS-ZM1. The co-incubation resulted in values of 79 ± 28% and 108 ± 60% for 0.5 and 1 mg mL^–1^ AGE-BSA. Unmodified BSA used at the same concentration did not alter serotonin release compared to the control (*p* > 0.05, data not shown). Similarly, FPS-ZM1 did not show any effects by itself (*p* > 0.05, data not shown). The treatment with 5, 50, 500 or 5000 μM lysine also did not change the extracellular serotonin concentration, leading to values of 148 ± 108%, 149 ± 86%, 108 ± 22% and 98 ± 22%, respectively (Fig. 2C).

### Proton secretion

**Fig. 4:**
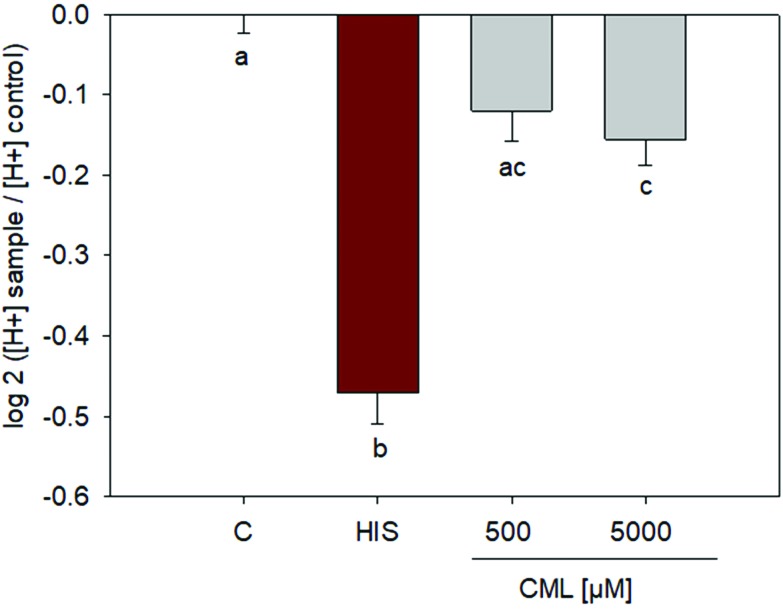
Proton secretory activity after treatment of HGT-1 cells with 500 μM or 5 mM CML; *n* = 3, data are shown as average with corresponding standard error of the mean, one-way ANOVA followed by Holm-Sidak *post hoc* test.

Incubation of HGT-1 cells with 500 μM CML did not induce any changes in proton secretion as calculated using the intracellular proton index (IPX), leading to an IPX value of –0.1203 ± 0.0382 (SEM) *vs.* control cells receiving buffer only. However, incubation with 5 mM CML resulted in a slight stimulation of proton secretion (Fig. 4), giving an IPX value of –0.1554 ± 0.0323 (*p* < 0.05, one-way ANOVA followed by Holm-Sidak *post hoc* test).

### CML concentration

The analysis of AGE-BSA and BSA by LC-MS/MS showed CML to be present in both modified and unmodified protein. A CML concentration of 10.2 ± 0.65 mmol mol^–1^ lysine (*n* = 3) was found in the AGE-BSA tested and a concentration of 0.15 ± 0.08 mmol mol^–1^ lysine (*n* = 3) was detected in the BSA sample.

## Discussion

Advanced glycation end products are ingested on a regular basis as part of a western diet. The advanced glycation end product CML and its precursor glyoxal are detectable in human plasma,^8^,^9^ both in increased concentrations in individuals suffering from diabetes.^9^,^35^ CML is predominantly found in a protein-linked state in plasma, but free CML has also been detected in concentrations up to 100 nM.^36^ As such, most *in vitro* studies into the effects of CML are carried out using either modified BSA or casein. However, recently, a conversion of free CML to protein-linked CML *in vivo* has been suggested in a study on the long-term exposure of rats on a high fat diet to large quantities of free CML.^37^ In addition, several studies showed comparable effects of free and protein-linked CML in different cell lines.^10^,^11^ As CML formation in the stomach has been reported to increase in the presence of catechols,^20^ and several Maillard reaction products have been indicated to exert influence on the mechanical activity of the gastric smooth muscle,^17^ we assessed the effect of CML on human parietal cells in culture, deriving from an adenocarcinoma of the stomach (HGT-1 cells). Furthermore, we have recently shown this cell line to be capable of serotonin biosynthesis and release upon stimulation.^25^ In our previous work on human SH-SY5Y cells, used as a model system of central serotonin, we could show treatment with 500 μM CML to increase serotonin release.^13^ Similarly, we have reported treatment with 500 μM CML to increase serotonin release from Caco-2 cells,^12^ indicating CML to influence serotonin release from peripheral sources, thus leading us to test whether CML also influenced gene expression of serotonin related genes and serotonin release from HGT-1 cells, representing cells from another peripheral site. First, we analysed the genome-wide impact of free CML and its precursor glyoxal on the gene expression of HGT-1 cells by microarray. Cluster analysis revealed both CML and glyoxal to influence the expression of serotonin receptors. As RAGE has been described to be overexpressed in gastric cancer cells compared to adjacent non-cancerous tissue,^38^ we analysed RAGE gene expression by qPCR. This experiment showed RAGE to be expressed by HGT-1 cells and glyoxal to alter its gene expression. CML, however, had no impact on RAGE gene expression. Next, we assessed the impact of glyoxal and CML on extracellular serotonin levels. This showed CML to have a strong impact on extracellular serotonin levels, while glyoxal showed no effect. However, the potential degradation of serotonin was not assessed, and whether the enzyme required for serotonin degradation, MAO-A, is expressed in HGT-1 cells is unknown. As a result, the total serotonin concentration may be underestimated, and it is unknown whether glyoxal or CML have any impact on a potential serotonin degradation, which may also result in an increased or decreased serotonin release compared to untreated control cells.

In order to test whether the increase in serotonin release after treatment with CML is related to RAGE, we carried out co-incubation experiments with the RAGE antagonist FPS-ZM1. This, however, unlike our experiments on Caco-2 cells,^12^ had no impact. As we used a longer incubation time compared to our Caco-2 study, and RAGE protein expression seems likely from our qPCR results, we next set out to elucidate whether a RAGE-mediated mechanism could be shown using a commercially available RAGE ligand, AGE-BSA, thus demonstrating that the cell system used in this study is capable of showing RAGE-related mechanisms. Unexpectedly, this experiment revealed the opposite effect on serotonin release compared to free CML. The treatment with AGE-BSA decreased serotonin release from HGT-1 cells and was reversible by co-incubation with the RAGE antagonist, indicating a RAGE-mediated pathway.

In order to exclude the impact of AGE-BSA to be caused by the protein itself regardless of the degree of modification, we analysed native BSA. BSA did not to have any influence on serotonin release, indicating, like the experiment using the RAGE antagonist, the effect of AGE-BSA is caused by the protein modification. Next, we determined the CML concentration in the modified and unmodified BSA. This showed, as expected, that the modified protein contained significantly higher amounts of CML. However, the highest concentration of free CML used, 5 mM, roughly corresponds to 1 mg mL^–1^ which is identical to the highest concentration of AGE-BSA used, thus making the CML concentration in the AGE-BSA incubation significantly lower than used in the incubations with free CML. However, a number of other modifications may be present in the AGE-BSA, which may also explain the opposite effect compared to the incubation with free CML. To test if the CML-induced serotonin release was caused by the amino acid itself rather than the lysine-modification, we incubated the cells with l-lysine in the same concentration range as CML. However, treatment with lysine could not induce the effects observed after incubation with CML. Recently, the amino acid l-arginine has been described to induce serotonin release from HGT-1 cells in a likely HTR3-mediated manner^25^ (unpublished data). In an attempt to clarify whether, similarly to l-Arg, the CML-induced serotonin release is HTR3-related, we carried out co-incubation experiments with the HTR3 inhibitor granisetron. In this case, the effect of 500 μM CML on serotonin release were counteracted, indicating a HTR3-related pathway. This pathway may be further analysed to elucidate the differences between AGE-BSA and free CML in terms of serotonin release. A study by Collison *et al.*^39^ described a link between RAGE activation and Ca^2+^ mobilization. Furthermore, a link between AGE, RAGE and Ca^2+^ homeostasis of the myocardium has been shown. This study by Petrova *et al.*^40^ showed myocardial overexpression of RAGE to reduce the systolic and diastolic Ca^2+^ concentration. Calcium ions and their role in neurotransmitter release are well described (see review by Augustine^41^) and differences in Ca^2+^ mobilization after treatment with free CML or AGE-BSA may be involved in the serotonin release induced by these compounds. Furthermore, HTR3 receptors are ion channels^42^ and, thus, form an exception compared to the G-protein coupled HTR receptors. In this study, we only investigated the involvement of HTR3, thus it cannot be excluded that other HTRs may also be involved and account for the discrepancy in observed serotonin release. SERT activity and potential 5-HT degradation were not analysed and should also be taken into consideration in further experiments as the 5-HT degradation product 5-hydroxyindole acetic acid has been reported to be biologically active. For instance, it has been demonstrated to decrease glucose uptake in a study on isolated rat soleus muscle.^43^ Differences in the intracellular signalling cascade utilizing mediators such as Ca^2+^, cAMP or pERK/ERK will also have to be addressed in future studies. However, we consider the effect of CML on HTR3-mediated pathways as physiologically relevant, in particular because an HTR3-related link between serotonin and gastric acid release has been suggested by Lai *et al.*^26^ In addition, the unmodified amino acid l-Lys has been reported to delay gastric emptying in a dose dependent manner in both rats and humans.^44^ As a result, we assessed the impact of CML on proton secretion. This showed the highest concentration tested, 5 mM to have a small, yet significant, stimulating effect, suggesting the influence of CML on factors involved in proton secretion to be further examined in future studies.

In conclusion, our current work shows AGE-BSA to reduce serotonin release in a likely RAGE-dependent manner. Furthermore, we show a likely HTR3-mediated, RAGE-independent impact of free CML on serotonin release in HGT-1 cells, in addition to showing an effect of free CML on cellular proton secretion.

## Abbreviations

AGE: Advanced glycation endproduct
BSA: Bovine serum albumin
CML: *N* ^ε^-Carboxymethyllysine
MRP: Maillard reaction product
RAGE: Receptor for advanced glycation end products
T/C: Treated over control

## Conflicts of interest

There are no conflicts to declare.
